# Augustus Woodruff Brown, D.D.S.

**Published:** 1895-09

**Authors:** 


### Obituary.

### AUGUSTUS WOODRUFF BROWN, D.D.S.

Augustus Woodruff Brown, D.D.S., died July 5 at his summer residence, in Manchester, Vt., aged ninety. He was born in Litchfield, Conn., and was thought to be the oldest dentist in America at the time of his death. He practised in New York City for half a century, and retired, with a fortune, fifteen years ago.

He was at first associated with Dr. Solomon Brown, his oldest brother, whose name was one of the best known in early dental literature. In their office, at 13 Park Place, New York City, the first dental society in the world was organized and the first dental journal planned. These were the American Society of Dental Surgeons, of which Eleazar Parmly was first president, and the *American Journal of Dental Science*, of which Dr. Solomon Brown was first editor.

Dr. Augustus W. Brown at one time had the most lucrative practice in New York, and was wildely known in social as well as professional circles.

One of the earliest honorary degrees of the Baltimore College of Dental Surgery was conferred upon him.

He married Miss Emma Mandeville, who with two daughters survives him. He had nine children, but none of his sons lived to manhood.

Dr. E. Parmly Brown, son of his brother Solomon, studied with him, and is the only member of the family now in the dental profession.

The funeral services were held at Manchester, and the interment was in the family vault in old Marble Cemetery, New York City.

